# Supplementary material for: The interactome of intact mitochondria by cross-linking mass spectrometry provides evidence for coexisting respiratory supercomplexes
Source: Mol Cell Proteomics. 2017 Dec 8;17(2):216–32. doi: 10.1074/mcp.RA117.000470 (PMC5795388; doi:10.1074/mcp.RA117.000470)
Supplement: Supplemental Data [file supp_RA117.000470_133922_0_supp_23978_fzffwf.zip › spectra_annotation/mito_spectra_annotation/356-1-4-1-3-1.pdf]

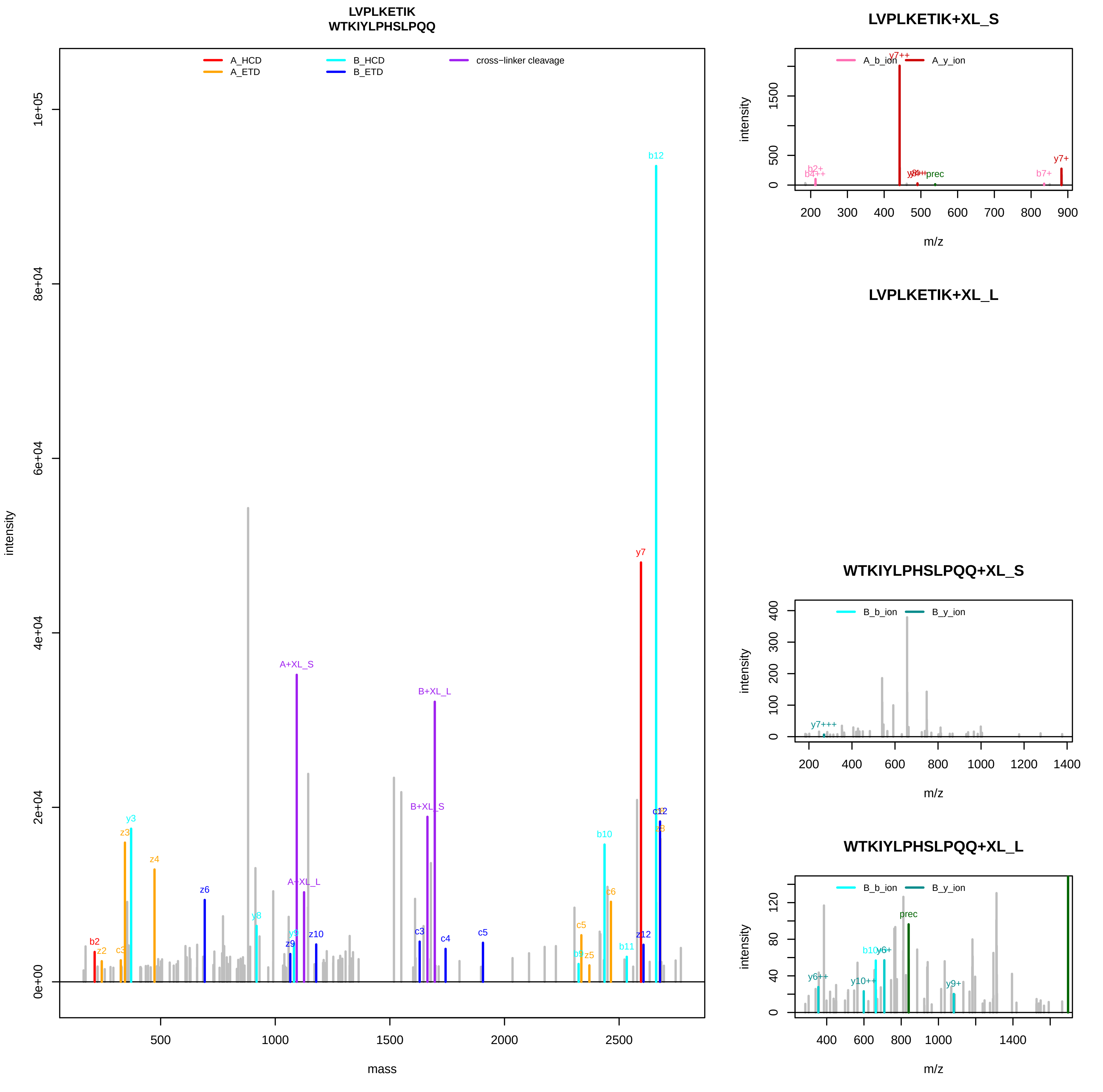

LVPLKETIK
WTKIYLPHSLPQQ
A_HCD
A_ETD
B_HCD
B_ETD
cross−linker cleavage
intensity
mass
b12
y7
A+XL_S
B+XL_L
B+XL_S
A+XL_L
y3
z3
z4
z6
y8
z9
z10
c3
c4
c5
b10
c6
c12
z12
b11
c5
z5
b9
b2
z2
LVPLKETIK+XL_S
A_b_ion
A_y_ion
y7++
y7+
b7+
prec
m/z
LVPLKETIK+XL_L
WTKIYLPHSLPQQ+XL_S
B_b_ion
B_y_ion
y7+++
WTKIYLPHSLPQQ+XL_L
prec
b10++
y6+
y6++
y10++
y9+
